# Complete Genome Sequence of *Paenibacillus larvae* MEX14, Isolated from Honey Bee Larvae from the Xochimilco Quarter in Mexico City

**DOI:** 10.1128/genomeA.00968-15

**Published:** 2015-08-27

**Authors:** D. Peréz de la Rosa, J. J. Pérez de la Rosa, R. Cossio-Bayugar, E. Miranda-Miranda, L. Lozano, M. A. Bravo-Díaz, M. K. Rocha-Martínez, B. Sachman-Ruiz

**Affiliations:** a Servicio Nacional de Sanidad, Inocuidad y Calidad Agroalimentaria, Morelos, México; b Centro Nacional de Investigación Disciplinaria en Parasitología Veterinaria del Instituto Nacional de Investigaciones Forestales Agrícolas y Pecuarias, Morelos, México; c Centro de Ciencias Genómicas, Universidad Nacional Autónoma de México, Cuernavaca, Morelos, México

## Abstract

*Paenibacillus larvae* strain MEX14 is a facultative anaerobic endospore-forming bacterium that infects *Apis mellifera* larvae. Strain MEX14 was isolated from domestic bee larvae collected in a backyard in Mexico City. The estimated genome size was determined to be 4.18 Mb, and it harbors 4,806 protein coding genes (CDSs).

## GENOME ANNOUNCEMENT

*Paenibacillus larvae* is the causative agent of American foulbrood (ABF) in practically all honey-producing countries, and Mexico, as the third largest exporter and fifth largest producer of honey in the world, is no exception. ABF has been reported in Mexico since 1994 (1), and a few officially acknowledged cases have been detected or isolated by the Mexican Agriculture Ministry.

*Paenibacillus larvae* strain MEX14 was obtained from a backyard in the Xochimilco quarter in México City in 2014 and isolated using the culture medium agar MYPGP (Mueller-Hinton broth, yeast extract, potassium phosphate, glucose, and pyruvate). DNA extraction was performed using a High Pure PCR template preparation kit (Roche). The complete genome was obtained with 454 GS FLX+ (Roche) sequencing technology*.*

A total of 380,502 raw reads were assembled using Newbler version 2.8. The obtained draft genome of *P. larvae* MEX14 contains 139 contigs with a total length of 4,185,110 bp and an *N*_50_ of 75,413. Genome sequence annotation was made by RAST (http://rast.nmpdr.org) (2). The resulting genome comprises 4,806 protein coding genes (CDSs), 52 rRNAs, and 71 tRNAs, with a G+C content of 44%. Genome analysis revealed that *P. larvae* MEX14 has high similarity to *Paenibacillus larvae* subsp. *larvae* DSM 25430 (an identity of 99.8%). Comparison of two *P. larvae* genomes provides evidence that the ABF causative agent contains a large number of virulent genes and multimodular enzymes, in addition to variability in size and a high number of genes (3), which suggest that making such a genome comparison with the MEX14 strain will provide new insights into the biology of this economically important pathogen of bees.

### Nucleotide sequence accession numbers.

This whole-genome shotgun project has been deposited at DDBJ/EMBL/GenBank under the accession no. LAWY00000000. The version described in this paper is version LAWY01000000.
